# Systems Biology: BAC to the Future

**DOI:** 10.1289/ehp.112-1277125

**Published:** 2004-08

**Authors:** Carol Potera

In a step forward into the future of gene expression research, molecular biologists and neurobiologists have joined forces to map the genes that control brain structure and neural circuits. The project, called the Gene Expression Nervous System Atlas, or GEN-SAT, maps mouse genes that are also present in the human genome as expressed in the central nervous system. According to project director Nathaniel Heintz, head of the Laboratory of Molecular Biology at The Rockefeller University, New York, GENSAT means that researchers studying degenerative conditions such as Parkinson disease can now have access to gene expression within the brain without having to do their own molecular genetics from scratch. Some unexpected insights have already come to light, giving neuroscientists new places to search for the roots of cognitive impairment.

GENSAT is sponsored by the National Institute of Neurological Disorders and Stroke (NINDS) and is based at The Rockefeller University, although prescreening of candidate genes is conducted by Tom Curran, chair of developmental neurobiology at St. Jude Children’s Research Hospital in Memphis, Tennessee. *In situ* hybridization is used to screen thousands of candidates to find genes that are active in the central nervous system. Of these, an advisory committee selects 250 genes each year for in-depth analysis by the Rockefeller group. Says Heintz, “Having an advisory committee means this research is done with consensus from many parts of the neuroscience community.”

Information gathered through the project is posted in a public database at **http://www.gensat.org/**. Started in 2003, the GENSAT database contains detailed information for 300 genes and is updated regularly. With the goal of analyzing 250 genes yearly, the project is planned to run for at least several more years, according to Heintz.

The main tools of GENSAT are bacterial artificial chromosomes (BACs), which are simple loops of bacterial DNA that reproduce outside the cell. BACs adeptly incorporate chunks of introduced DNA from other species, which are preserved and duplicated along with the BACs. The Human Genome Project relied on BACs to help map the human genome.

To measure gene activity and patterns of gene expression in the brain, the GEN-SAT team inserts a reporter gene for enhanced green fluorescent protein into each BAC. When genes are active, the enhanced green fluorescent protein glows bright green. Each BAC is then inserted into eggs harvested from mice, and the eggs are implanted into foster mothers.

The resulting offspring carry the BAC throughout their bodies in all of the cells that express the corresponding gene. Groups of mice are sacrificed at three time points—two of which correspond to critical periods of human central nervous system development—and their brains and spinal cords are analyzed. Mapping gene activity at three different points reveals how the cells migrate and interact.

The first samples are taken when the mouse embryos are 15 days old, which corresponds to the sixth to seventh month of human gestation. “During this period the cortex forms, and defects that lead to malformations occur,” explains project codirector Mary Beth Hatten, head of the Laboratory of Developmental Neurobiology at Rockefeller. The second time point, at 7 days after birth, is equivalent to 6–8 months of age in humans. At this age, interconnections form in the cerebellum, which controls movement, and in the hippocampus, which controls short-term memory. The final observations are made on adult mouse brains at age 7 months, which are similar to those of 30-year-old humans.

Findings published in the 30 October 2003 issue of *Nature* reveal some of the surprising connections the GENSAT project is uncovering. For example, people with DiGeorge syndrome, a congenital condition marked by heart defects and learning disorders, lack a gene called *Gscl*. Heintz, Hatten, and other GENSAT researchers discovered that *Gscl* is produced by neurons in the interpeduncular nucleus, the brain region that also regulates rapid-eye-movement sleep. Another finding reported in this paper relates to the striatum, which degenerates in patients with Parkinson disease. In end-stage Parkinson disease, up to 95% of so-called spiny neurons are lost. Until recently, the striatum had been the only place where spiny neurons were found, says Hatten. Yet, the BAC method identified vectors that can be used to separately analyze spiny neurons that project to the substantia nigra and the globus pallidus.

The GENSAT methods can also monitor the effects of environmental toxicants, such as lead, on brain development. “You can expose the BAC mice to any environmental condition you want, to see how the migration and maturation of neurons changes,” says Hatten.

“The tools and mouse lines provided by this project allow the neuroscience community to perform detailed studies of each gene,” says Laura Mamounas, the GEN-SAT project officer at the NINDS. “GEN-SAT also may serve as a model for future gene expression projects.”

Indeed, BAC mice can be used to screen gene activity in other organs. The BAC mice are made available to other researchers who are interested in performing systematic studies of gene expression. Scientists in other specialties are “just starting to bootstrap our efforts to get their particular information,” says Heintz.

## Figures and Tables

**Figure:**
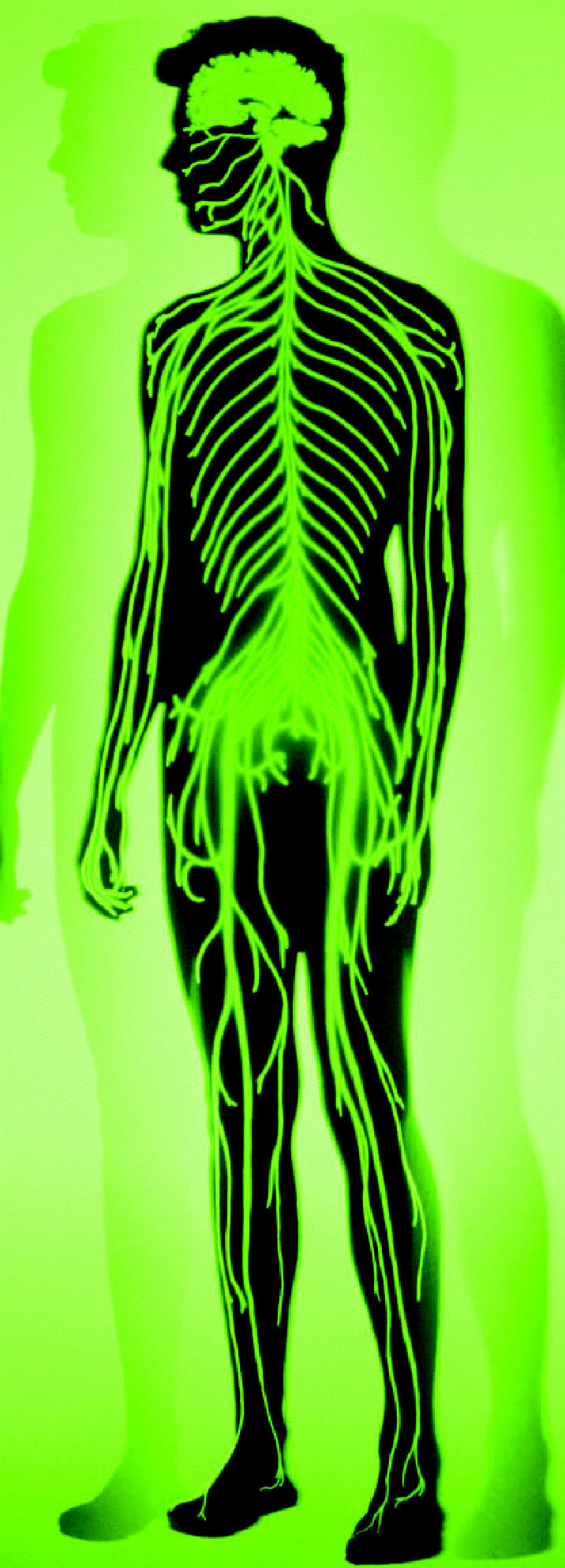
**Mr. Greengenes.** The GENSAT project uses enhanced green fluorescent protein to map mouse genes that are also present in humans and expressed in the central nervous system.

